# Retraction: MicroRNAs, long non-coding RNAs, and circular RNAs and gynecological cancers: focus on metastasis

**DOI:** 10.3389/fonc.2026.1967877

**Published:** 2026-09-01

**Authors:** Frontiers Editorial Office

The journal retracts the 03 of October 2023 article cited above.

Following publication, concerns were raised that a large a large number of citations refer to retracted articles. An investigation was conducted in accordance with Frontiers’ policies.

It was found that the complaints were valid and that the article does not meet the standards of editorial and scientific soundness for Frontiers in Oncology; therefore, the article has been retracted.

This retraction was approved by the Chief Editors of Frontiers in Oncology and the Chief Executive Editor of Frontiers. Maryam Mahjoubin-Tehran does not agree to this retraction. The rest of the authors did not respond to correspondence regarding this retraction.

Frontiers would like to thank the Problematic Paper Screener and PubPeer users (https://pubpeer.com/publications/8033BE0DEBC4DDCEF56B140A91F426) for bringing the published article to our attention.

